# *Toxoplasma gondii* seroprevalence and genotype diversity in select wildlife species from the southeastern United States

**DOI:** 10.1186/s13071-017-2456-2

**Published:** 2017-10-23

**Authors:** Richard W. Gerhold, Pooja Saraf, Alycia Chapman, Xuan Zou, Graham Hickling, William H. Stiver, Allan Houston, Marcy Souza, Chunlei Su

**Affiliations:** 1 Department of Biomedical and Diagnostic Sciences, College of Veterinary Medicine, University of Tennessee Institute of Agriculture, Knoxville, TN 37996 USA; 2 Center for Wildlife Health, Department of Forestry, Wildlife, and Fisheries, University of Tennessee Institute of Agriculture, Knoxville, TN 37996 USA; 3 Department of Microbiology, The University of Tennessee, Knoxville, TN 37996 USA; 4 Great Smoky Mountains National Park, 107 Park Headquarters Road, Gatlinburg, TN 37738 USA; 5 Ames Plantation, 4275 Buford Ellington Rd, Grand Junction, TN 38039 USA

**Keywords:** *Toxoplasma gondii*, Toxoplasmosis, Wildlife

## Abstract

**Background:**

*Toxoplasma gondii* is a widespread protozoan parasite that infects humans and other animals. Previous studies indicate some genotypes of *T. gondii* are more frequently isolated in wildlife than agricultural animals, suggesting a wild/feral animal diversity model. To determine seroprevalence and genetic diversity of *T. gondii* in southeastern US wildlife, we collected sera from 471 wild animals, including 453 mammals and 18 birds, between 2011 and 2014. These serum samples were assayed for *T. gondii* infection using the modified agglutination test (MAT). Heart or tongue tissues from 66 seropositive animals were bioassayed in mice and 19 isolates were obtained. The isolated parasites were genotyped by the polymerase chain reaction-restriction fragment length polymorphism (PCR-RFLP) method employing 10 genetic markers.

**Results:**

One hundred and ninety-six of 471 samples (41.6%) had a titer ≥1:32 and were considered positive for *T. gondii* infection. Of 453 mammals, 195 (43%) were seropositive, whereas only one (5.6%) of 18 birds was seropositive. The seroprevalence in mammals was significantly higher than in the birds. Mammalian hosts with adequate samples size (≥ 20) comprised white-tailed deer (*n* = 241), feral hogs (*n* = 100), raccoons (*n* = 34) and coyotes (*n* = 22), with seroprevalences of 41.0%, 51.0%, 50.0% and 72.7%, respectively. Coyotes had significantly higher seroprevalence than the white-tailed deer. Genotyping revealed five distinct genotypes, including the ToxoDB PCR-RFLP genotype #5 (a.k.a type 12) for 15 isolates, genotype #3 (a.k.a. type II) for 1 isolate, and genotypes #154, #167 and #216, each for 1 isolate. The results showed moderate to high infection rates of *T. gondii* in white-tailed deer, feral hogs, raccoons and coyotes. Genotyping results indicated limited genetic diversity and a dominance of genotype #5, which has been reported as a major type in wildlife in North America.

**Conclusions:**

We conclude that *T. gondii* infection is common in game animals (white-tailed deer and feral hogs) in the southeastern US, which may pose a food safety risk to humans. Further research is necessary to understand *T. gondii* transmission from wildlife to farm animals and humans.

## Background

Toxoplasmosis, caused by *Toxoplasma gondii*, is zoonotic and considered a leading cause of human morbidity attributed to food borne illness in the United States [1], and it is estimated that one-third of the world’s population is infected by this pathogen [2]. Women infected with *T. gondii* during pregnancy can have variable consequences including pregnancy complications, stillbirths and abortions. In immunocompromised patients, such as those with AIDS, encephalitis may occur, which is often fatal [2]. Toxoplasmosis is one of five neglected parasitic infections that have been targeted by the Centers for Disease Control and Prevention (CDC) for public health action. Infection with *T. gondii* can occur by ingestion of microscopic oocysts in contaminated food or water, or by ingestion of tissue cysts in undercooked or raw meat [2, 3], making it an important foodborne zoonotic pathogens.

*Toxoplasma gondii* infection occurs in many species of wild mammals and birds, particularly those that are carnivorous or ground dwelling. Clinical toxoplasmosis occurs in a wide variety of US wildlife, including threatened and endangered terrestrial and marine mammals and birds [4, 5]. Epidemiology studies of white-tailed deer populations have reported seroprevalence from 30% to 76% in areas including Pennsylvania, Minnesota, Mississippi, New Jersey, Iowa and Ohio [6–10]. A range of seroprevalence (15–84%) was observed in raccoons from Iowa, New Jersey, Ohio, Kansas, Illinois, Florida, Pennsylvania, Virginia and Wisconsin [11–14]. A high seroprevalence in red and gray foxes (85.9%) was reported in Kentucky, Indiana, Michigan and Ohio [15, 16] and wild hogs from California and black bears from Pennsylvania also show seroprevalence of 17% and 75–80%, respectively [8, 17]. Antibodies against *T. gondii* (7–17%) were detected in wolves from remote areas in Alaska [18, 19]. Genotyping of wildlife isolates suggests that wild animals maintain a much greater diversity of *T. gondii* genotypes than agricultural animals [20–22]. There is no reported association between *T. gondii* genotypes and disease manifestation, but some evidence suggests a relationship. For example, in South America, where wild animal populations are more dominant, severe cases of human toxoplasmosis were reported even in immunocompetent adults [23–26], and the majority of these infections were attributed to unique genotypes. Recent studies have reported the presence of numerous genotypes in wildlife populations in North America. Currently, ToxoDB PCR-RFLP genotypes #4 and #5, also known as type 12, are recognized as the dominant type in North America wildlife [20, 21]. It is likely that some of these *T. gondii* strains from wildlife are highly virulent, posing a potential wildlife health risk and a higher risk for severe toxoplasmosis if transmitted in human populations.

The role of wildlife in the transmission of *T. gondii* demands increased efforts to catalog the major sources of human *T. gondii* infection. Continued characterization is critical to understanding the potential risks of *T. gondii* to wildlife populations and its zoonotic implications. Seroprevalence and genotyping data from the southeast region of the United States have been insufficient to determine the pattern of *T. gondii* transmission in the area. Hence, in this study, we focused on determining seroprevalence and characterizing strains isolated from wildlife in this region.

## Methods

Serum with or without corresponding fresh heart or tongue tissue samples was collected from hunter-killed, road killed, nuisance killed (i.e. feral hogs), or research collected animals from multiple southeastern states (Table 1). Tissue samples were refrigerated until serological screening was completed.

**Table 1:** Seroprevalence of *T. gondii* in wildlife by county and State in the southeastern USA

State of origin	County/Site	Species	Seropositive/ total	Seroprevalence (%)
Tennessee (*n* = 309; 293 mammals, 16 birds)	Loudon	white-tailed deer	5/9	55.6
	Knox	opossum	1/2	50.0
		woodchuck	1/4	25.0
		mink	1/1	100
		raccoon	5/12	41.7
		gray fox	2/3	66.7
		opossum	2/3	66.7
		pigeon	1/1	100
		other^a^	0/32	0
	Coffee	white-tailed deer	1/3	33.3
		gray squirrel	0/9	0
	Ames Plant	coyote	11/17	54.1
		white-tailed deer	40/77	51.9
		raccoon	5/8	62.5
		opossum	3/7	42.9
	GSMNP	black bear	0/1	0
		feral hog	11/27	40.7
	Kingston	raccoon	7/13	53.8
	Oak Ridge	white-tailed deer	18/64	28.1
	AEDC, Decherd	white-tailed deer	2/14	14.2
	Jefferson	raccoon	0/1	0
	Roane	mink	0/1	0
South Carolina (*n* = 74)	Laurens	white-tailed deer	33/74	44.6
North Carolina (*n* = 74)	GSMNP	feral hog	40/73	54.8
		black bear	0/1	0
Georgia (*n* = 6)	Jefferson	gray fox	0/1	0
	Putnam	coyote	5/5	100
Alabama (*n* = 4)	Brent	woodchuck	0/1	0
	Hale	eastern cottontail	0/1	0
		armadillo	0/1	0
		gray squirrel	0/1	0
Kentucky (*n* = 4)	Perry	elk	2/3	66.7
	Knott	elk	0/1	0

Overall prevalence = 196/471 (41.6%)

*Abbreviations*: *GSMNP *Great Smoky Mountain National Park, *AEDC* Arnold Engineering development complex

^a^Total 32 samples (15 mammals, 17 birds): one sample from each of the following wildlife: American crow, American robin, beaver, belted kingfisher, chickadee, chimney swift, fox squirrel, gray catbird, hermit thrush, house sparrow, oven bird, pileated woodpecker, rock pigeon, tufted titmice and turkey vulture. Two samples from: eastern chipmunk, blue jay and mourning dove. Four samples from: gray squirrel. Seven samples from eastern cottontail

Screening for *T. gondii* was performed at the clinical parasitology laboratory at the University of Tennessee, College of Veterinary Medicine using the MAT test as previously described [27, 28]. This assay is used to detect anti-*T. gondii* antibodies in blood, serum and other bodily fluids from a wide variety of wildlife and domestic species. Animals were considered *Toxoplasma* positive if IgG antibodies were detected at ≥ 1:32 dilution on MAT. Three to 5 g of heart or tongue tissue from some seropositive hosts were processed and used in bioassays of mice to propagate *T. gondii* [29]. To facilitate isolation of *T. gondii*, mice were treated with 15 μl/ml dexamethasone in drinking water at the time of inoculation of processed animal tissues. Mice showing clinical signs of infection (roughed fur and lethargic) were terminated, peritoneal lavage are collected and inoculated to cell culture to expand the parasites. All nonclinical mice were terminated on day 14 post-inoculation, peritoneal lavage was collected and inoculated to cell culture. Isolated *T. gondii* strains were genotyped by multiplex multilocus nested PCR-RFLP (Mn-PCR-RFLP) employing 10 genetic markers [30].

To compare seroprevalence of different populations, data analysis was performed using statistical software SAS version 9.4. Chi-square tests were conducted to determine if there was statistically significant difference among different sampling groups. Logistic regression was used to compute the odds ratios of infection among different groups. Association of serum MAT titer with success of isolating *T. gondii* in bioassay was assessed by linear regression analysis using SAS GLM procedure (SAS 9.4).

## Results

### Seroprevalence of *T. gondii*

A total of 471 serum/plasma samples were collected from 31 wildlife species (16 mammal and 15 bird species) between 2011 and 2014 (Table 2). Samples originated in six southeast states, comprising Alabama, Georgia, Kentucky, North Carolina, South Carolina and Tennessee (Table 1). From 471 samples, 41.6% (196/471) had MAT titers ≥ 1:32 and were considered positive for *T. gondii* infection (Tables 1, 2). Nine mammalian (white-tailed deer, opossum, raccoon, coyote, feral hog, woodchuck, elk, gray fox and mink) and 1 bird species (rock pigeon) collected from five southeastern states had seropositive individuals (Tables 1, 2). Mammal hosts with samples size ≥ 10 individuals comprised white-tailed deer (*n* = 241), feral hogs (*n* = 100), raccoons (*n* = 34), coyotes (*n* = 22), opossum (*n* = 12) and gray squirrels (*n* = 14) and had seroprevalences of 41%, 51%, 50%, 72.7%, 50% and 0%, respectively.

**Table 2:** Seroprevalence of *T. gondii* in southeastern wildlife species in USA

Host	No. of samples	MAT titers					Seroprevalence (%)
		< 1:32	1:32–1:128	1:256–1:1024	1:2048–1:8192	> 1:8192	
Mammals (*n* = 453, seropositive 195)							
White-tailed deer	241	142	59	11	11	18	41.0
Feral hog	100	49	33	13	4	1	51.0
Raccoon	34	17	6	7	0	4	50.0
Coyote	22	6	5	4	4	3	72.7
Woodchuck	5	4	1	0	0	0	20.0
Elk	4	2	1	1	0	0	50.0
Gray fox	4	2	2	0	0	0	50.0
Mink	2	1	0	0	1	0	50.0
Opossum	12	6	3	2	1	0	50.0
Gray squirrel	14	14	0	0	0	0	0
Eastern cottontail	8	8	0	0	0	0	0
Black bear	2	2	0	0	0	0	0
Armadillo	1	1	0	0	0	0	0
Beaver	1	1	0	0	0	0	0
Eastern chipmunk	2	2	0	0	0	0	0
Fox squirrel	1	1	0	0	0	0	0
Birds (*n* = 18, seropositive 1)							
Blue jay	2	2	0	0	0	0	0
Mourning dove	2	2	0	0	0	0	0
American crow	1	1	0	0	0	0	0
American robin	1	1	0	0	0	0	0
Belted kingfisher	1	1	0	0	0	0	0
Chickadee	1	1	0	0	0	0	0
Chimney swift	1	1	0	0	0	0	0
Gray catbird	1	1	0	0	0	0	0
Hermit thrush	1	1	0	0	0	0	0
House sparrow	1	1	0	0	0	0	0
Oven bird	1	1	0	0	0	0	0
Pileated woodpecker	1	1	0	0	0	0	0
Rock pigeon	2	1	1	0	0	0	50.0
Tufted titmice	1	1	0	0	0	0	0
Turkey vulture	1	1	0	0	0	0	0
Total	471	275	111	38	21	26	41.6% (196/471)

In Tennessee, 309 serum samples from 29 animal species were collected and tested from 10 counties/sites (Table 1). Overall, 37.5% (116/309) were positive for *T. gondii* infection. In South Carolina, 74 serum samples from white-tailed deer in Laurens County were tested, with 44.5% (33/74) seropositive. In North Carolina, 74 serum samples (73 from feral hogs, 1 from a black bear) were collected from the GSMNP (Table 1), 54.1% (40/74) positive. For feral hogs, 54.8% (40/73) were positive to *T. gondii* infection. In Georgia, 6 serum samples were collected from 5 coyotes and 1 gray fox in Jefferson and Putnam counties (Table 1). The 5 samples from coyotes in Putnam County were all seropositive. Four serum samples from 4 animal species in Alabama were all negative (Table 1). Two of 4 samples from elk in Kentucky were positive (50%).

### Comparison of seroprevalence in different wildlife hosts and geographical locations

Seroprevalence in mammals was 39.2% (195/453), which was significantly higher than in birds (5.6%, 1/18) (Chi-square test: *χ* ^2^ = 6.10, *df* = 1, *P* = 0.014; Odds ratio: 12.84; 95% CI: 1.695–97.26). Among the mammal populations with sample size ≥ 20, including Tennessee (115/293), South Carolina (33/74) and North Carolina (40/74), there was no statistically significant difference in seroprevalence (Chi-square test: *χ* ^2^ = 5.36, *df* = 2, *P* = 0.068). Comparison of seroprevalence for white-tailed deer and feral hogs that had sample size ≥ 20 in different geographical locations was performed. Seroprevalence rates in white-tailed deer from Ames Plantation (Tennessee), Oak Ridge (Tennessee) and Laurens (South Carolina) were 51.9, 28.1 and 44.6%, respectively (Table 1). White-tailed deer from Ames Plantation and Laurens had significantly higher odds of being positive than those in Oak Ridge (Chi-square test: *χ* ^2^ = 8.14, *df* = 2, *P* = 0.017), with Laurens *vs* Oak Ridge, odds ratio 2.057 (95% CI: 1.009–4.192); Ames Plantation *vs* Oak Ridge, odds ratio 2.763 (95% CI: 1.365–5.590). Seroprevalence rates in white-tailed deer from Ames Plantation and Laurens were not significantly different (odds ratio 1.343, 95% CI: 0.708–2.548). Seroprevalence rates in feral hogs from GSMNP Tennessee and GSMNP North Carolina were 40.7 and 54.8%, respectively (Table 1). There was no statistically significant difference between the two groups (Chi-square test: *χ* ^2^ = 1.54, *df* = 1, *P* = 0.215; Odds ratio: 1.763; 95% CI: 0.720–4.317).

Comparison of seroprevalence was also conducted for wildlife species that had sample size ≥ 20 regardless of geographical locations. These species included white-tailed deer (*n* = 241), feral hogs (*n* = 100), raccoons (*n* = 34) and coyotes (*n* = 22), which had seroprevalence rates of 41.0, 51.0, 50.0, and 72.7%, respectively (Table 2). Significant difference was detected (Chi-square test: *χ* ^2^ = 9.24, *df* = 3, *P* = 0.026), with coyotes having a significantly higher infection rate than white-tailed deer (odds ratio 3.825, 95% CI 1.446–10.117). No differences were detected among other species.

### Isolation and genotyping of *T. gondii* strains

Tissue (hearts and tongues) from 66 seropositive wildlife samples were bioassayed in mice. These samples comprised: 33 from white-tailed deer, 11 from feral hogs, 8 from raccoons, 8 from coyotes, 2 from elks, 2 from opossums, 1 from mink and 1 from gray fox. Nineteen *T. gondii* isolates were obtained by bioassay (13 from white-tailed deer, 3 from feral hogs, 2 from coyotes and 1 from a mink) (Table 3). For tissue samples with MAT titers of 32, 128, 512, 2048, 4096 and ≥ 8192, the rates of obtaining *T. gondii* isolates in bioassay were 0, 15, 12.5, 20, 66.7 and 62.5%, respectively. There was a significant correlation between MAT titers and the success rates of bioassay (GLM linear regression coefficient *r* = 0.88, *P* = 0.021).

**Table 3:** Isolation of *T. gondii* by bioassay in mice

Sample ID	Host	Location	Date sample collected	MAT titer	Days between collection and inoculation	Positive in cell culture/mice used^a^	Isolate ID
17	wtd	TN	10/8/2011	≥ 8192	6	3/3	TgMnkTn17
40	wtd	SC	10/15/2011	≥ 8192	16	2/3	TgWtdSc40
43	wtd	SC	10/15/2011	≥ 8192	16	2/3	TgWtdSc43
60	wtd	SC	10/15/2011	≥ 8192	16	3/3	TgWtdSc60
78	wtd	SC	11/13/2011	≥ 8192	9	2/2	TgWtdSc78
88	wtd	SC	11/13/2011	≥ 8192	9	2/2	TgWtdSc88
98	wtd	SC	11/13/2011	2048	9	2/2	TgWtdSc98
99	wtd	SC	11/13/2011	2048	9	2/2	TgWtdSc99
110	wtd	SC	11/13/2011	≥ 8192	9	2/2	TgWtdSc110
113	wtd	SC	11/13/2011	≥ 8192	9	2/2	TgWtdSc113
122	coyote	TN	1/21/2012	512	12	1/1	TgCyTn122
142	coyote	TN	2/2/2012	4096	14	2/2	TgCyTn142
194	feral hog.	NC	1/28/2013	4096	16	2/3	TgHogNc194
227	feral hog	NC	4/9/2013	128	16	2/3	TgHogNc227
335	wtd	TN	11/12/2013	128	9	1/2	TgWtdTn335
372	wtd	TN	12/9/2013	≥ 8192	4	2/2	TgWtdTn372
387	wtd	TN	12/9/2013	≥ 8192	4	2/2	TgWtdTn387
399	wtd	TN	12/15/2013	2048	8	1/2	TgWtdTn399
452	feral hog	NC	1/16/2014	128	18	2/3	TgHogNc452

*Abbreviations*: *NC *North Carolina, *SC* South Carolina, *TN* Tennessee, *wtd* white-tailed deer

^a^Number of mice from which *T. gondii* was successfully isolated and expanded in cell culture versus total number of mice used for bioassay

The 19 *T. gondii* isolates were genotyped by the 10 PCR-RFLP markers (Table 4). Five distinct genotypes were identified: ToxoDB PCR-RFLP genotype #5 (15 isolates), #3 (1 isolate), #154 (1 isolate), #167 (1 isolate) and #216 (1 isolate). Of the 13 isolates obtained from white-tailed deer, 9 were from South Carolina and 4 from Tennessee.

**Table 4:** Genotyping of *T. gondii* isolates from wildlife

ID	Host	Location	SAG1	5′-3′ SAG2	alt. SAG2	SAG3	BTUB	GRA6	c22-8	c29-2	L358	PK1	Apico	Genotype
TgWtdSc40	wtd	SC	u-1	II	II	II	II	II	II	II	I	II	I	#5
TgWtdSc43	wtd	SC	u-1	II	II	II	II	II	II	II	I	II	I	#5
TgWtdSc60	wtd	SC	u-1	II	II	II	II	II	II	II	I	II	I	#5
TgWtdSc78	wtd	SC	u-1	II	II	II	II	II	II	II	I	II	I	#5
TgWtdSc99	wtd	SC	u-1	II	II	II	II	II	II	II	I	II	I	#5
TgWtdSc113	wtd	SC	u-1	II	II	II	II	II	II	II	I	II	I	#5
TgWtdSc88	wtd	SC	I	II	II	III	II	II	II	u-1	III	II	I	#154
TgWtdSc98	wtd	SC	II or III	I	I	I	I	I	I	III	III	III	III	#167
TgWtdSc110	wtd	SC	I	I	I	III	III	I	III	III	III	I	III	#216
TgHogNc194	feral hog	NC	u-1	II	II	II	II	II	II	nd	I	nd	nd	#5
TgHogNc227	feral hog	NC	u-1	II	II	II	II	II	II	nd	I	nd	nd	#5
TgHogNc452	feral hog	NC	u-1	II	II	II	II	II	II	II	I	II	I	#5
TgMnkTn17	mink	TN	II or III	II	II	II	II	II	II	II	II	II	I	#3
TgCyTn122	coyote	TN	u-1	II	II	II	II	II	II	II	I	II	I	#5
TgCyTn142	coyote	TN	u-1	II	II	II	II	II	II	II	I	II	I	#5
TgWtdTn335	wtd	TN	u-1	II	II	II	II	II	II	II	I	II	I	#5
TgWtdTn372	wtd	TN	u-1	II	II	II	II	II	II	II	I	II	I	#5
TgWtdTn387	wtd	TN	u-1	II	II	II	II	II	II	II	I	II	I	#5
TgWtdTn399	wtd	TN	u-1	II	II	II	II	II	II	II	I	II	I	#5

*Abbreviations*: *NC *North Carolina, *nd* no data, *SC* South Carolina, *TN* Tennessee, *wtd* white tailed deer

## Discussion

The present study demonstrates that *T. gondii* infection is widespread in wild mammals from the southeastern United States. We collected sera from 471 wild animals (453 mammals and 18 birds) between 2011 and 2014. Overall, 41.6% were positive for *T. gondii* infection, however, only one of the 18 birds was seropositive (Table 2). The seroprevalence in mammals was significantly higher than in the birds. Among the most frequently sampled mammal species (white-tailed deer, feral hog, raccoon and coyote, *n* ≥ 20 each), seroprevalence varies from 41% to 72.7%, with that for coyote significantly higher than for white-tailed deer (Table 2), which supports the general idea that carnivores have higher infection rates than herbivores.

Among the three geographical locations with mammal samples size ≥ 20, Tennessee (*n* = 293), South Carolina (*n* = 74) and North Carolina (*n* = 74), seroprevalence rates varied from 39.2% to 54.1%, however, there was no statistically significant difference. Among white-tailed deer populations from three different locations, Ames Plantation (Tennessee), Oak Ridge (Tennessee), and Laurens (South Carolina), seroprevalence in Oak Ridge was significantly lower than the other two populations, which warrants future studies to understand what factors contribute to such a difference. Seroprevalence rates in feral hogs from North Carolina and Tennessee sides of the Great Smoky Mountains National Park were, in general, not significantly different, which is expected given the similar environment.

In this study, the success rate of bioassay was 28.8% (19/66). Efficiency of bioassay can be affected by many factors, such as how long the tissue samples were stored before inoculated to mice, the amount of tissues used, and the type of tissues used. In addition, tissue cysts may not evenly distribute in the muscle or brain tissues of infected animals, and successfully obtaining cysts variable between samples. We did an analysis of MAT titers *vs* success rates in bioassay; it showed a positive correlation, suggesting higher titers may have higher parasite load in the tissues.

Genotype #5 (a.k.a. type 12) is the most common circulating genotype in wildlife in this region of the US, which is in agreement with previous studies reporting the prevalence of genotype #5 in white-tailed deer populations [20, 31]. Genotypes #156 and #167 have been previously reported from goats in the USA [32]. Two isolates from coyote (TgWtdTn122 and TgWtdTn142) and 1 mink isolate (TgMnkTn17) obtained from Tennessee, belong to genotype #5 and #3, respectively. Genotype #3 (type II) of *T. gondii* is the most dominant lineage distributed globally. Furthermore, the 2 feral hog isolates (TgHogNc194 and TgHogNc227) from North Carolina also belonged to genotype #5, which is commonly distributed in North America [31]. We were unable to assess the virulence of *T. gondii* strains in mice during the bioassay, as mice were treated with dexamethasone to suppress their immune responses and the experiments were terminated on day 14 post-infection.

## Conclusions

In addition to the commonly observed genotypes, we also isolated several non-clonal types circulating in sampled populations. This is of interest, as previous epidemiological studies have reported a link between the prevalence of non-clonal genotypes and cases of congenital ocular and severe disseminated toxoplasmosis in areas such as Brazil [33]. White-tailed deer is one of the dominant wildlife species found in North America and venison a common game meat. Thus, the high seroprevalence in this species indicates that deer could serve as a potential source of human infection. Hence, people consuming wild venison should be advised to cook the meat properly and use caution while handling the raw meat. Future genotyping and seroprevalence studies in wildlife hosts, and analysis of their role in the transmission cycle, will increase the understanding of risks associated with *T. gondii* in human populations.

## Abbreviations

MAT: Modified agglutination test
PCR-RFLP: Polymerase chain reaction-restriction fragment length polymorphism
